# Predictors of a placebo response in patients with hand osteoarthritis: post-hoc analysis of two randomized controlled trials

**DOI:** 10.1186/s12891-021-04089-9

**Published:** 2021-03-04

**Authors:** Jin Kyun Park, Se Han Ahn, Kichul Shin, Yun Jong Lee, Yeong Wook Song, Eun Bong Lee

**Affiliations:** 1 Department of Internal Medicine, Division of Rheumatology, Seoul National University College of Medicine, 101 Daehak-ro, Jong no-gu, Seoul, 03080 South Korea; 2 Department of Internal Medicine, Seoul Metropolitan Government-Seoul National University Boramae Medical Center, Seoul, South Korea; 3 Department of Internal Medicine, Division of Rheumatology, Seoul National University Bundang Hospital, Seongnam-si, Gyeonggi-do South Korea; 4 Department of Molecular Medicine and Biopharmaceutical Sciences, Graduate School of Convergence Science and Technology, Seoul National University, Seoul, South Korea

**Keywords:** Osteoarthritis, Hand, Placebo

## Abstract

**Background:**

Placebo can have a significant therapeutic effect in patients with hand osteoarthritis (OA). This aim of the study is to identify factors associated with a clinically meaningful placebo response in patients with hand OA.

**Methods:**

This post-hoc analysis of two double-blind, placebo-controlled, randomized trials (RCTs) investigating the efficacy of GCSB-5 or diacerein as treatments for hand OA analyzed the efficacy of a placebo. Clinical and laboratory factors associated with a clinically meaningful response, defined as an improvement in the Australian/Canadian Osteoarthritis Hand Index (AUSCAN) pain score > 10 at 4 weeks relative to baseline, were identified.

**Results:**

The mean improvement in the AUSCAN pain score was − 6.0 ± 20.3, with marked variation between 143 hand OA patients (range: − 76.4 to 33.2). A clinically meaningful improvement was observed in 54 (37.8%) patients. Placebo responders had worse AUSCAN pain scores (55.7 ± 19.7 vs. 43.6 ± 21.6, *p* = 0.001) and a worse AUSCAN stiffness (68.2 ± 20.5 vs. 57.5 ± 24.5, *p* = 0.008) at baseline than non-responders. Improvements in pain correlated with the baseline pain level (Pearson *r* = − 427, *p* < 0.001). Structural joint changes such as tender, swollen, enlarged, or deformed joint counts did not differ between placebo responders and non-responders. In a multivariable analysis, only baseline AUSCAN pain was associated with a clinically meaningful placebo response (OR: 1.054, 95% CI [1.019–1.089], *p* = 0.002).

**Conclusions:**

High levels of pain at baseline are predictive of a clinically meaningful placebo response in patients with hand OA. Further studies are needed to optimize and utilize the benefit of placebo responses in patients with hand OA.

**Supplementary Information:**

The online version contains supplementary material available at 10.1186/s12891-021-04089-9.

## Background

Osteoarthritis (OA) of the hands is common in middle-aged and elderly populations, especially women [1]. The marked disability and reduced quality of life caused by the disease are comparable with those caused by rheumatoid arthritis (RA) [2, 3]. Pain can be especially debilitating in patients with erosive hand OA, which is characterized by painful swelling, and joint inflammation as well as the subchondral bone erosions and marginal osteophyte formation on radiographic images. The main therapeutic approach to hand OA is to control symptoms by using a combination of non-pharmacological and pharmacological interventions [4]; this is because, unlike for RA, there are no effective disease-modifying osteoarthritis drugs (DMOADs). To date, oral non-steroidal anti-inflammatory drugs (NSAIDs), acetaminophen, or opioid-based analgesics constitute the mainstay of treatment targeting pain control. Inflammatory cytokines such as interleukin-1 (IL-1), tumor necrosis factor alpha (TNF-α) may contribute to the degeneration of articular cartilage matrix [5]. Therefore, treatment targeting inflammation and pro-inflammatory cytokines were attempted. In a recent study, a short-term treatment with low dose corticosteroid improved pain and signs of inflammation in patients who experience a flare-up of hand OA [6]. However, they and other medications, including anti-tumor necrosis factor inhibitors and anti-interleukin-1 antibody, show only minimal to moderate effect sizes, emphasizing an unmet need for better treatment modalities for hand OA [7–9].

A previous randomized clinical placebo-controlled trial (RCT) involving patients with hand OA reported that 30.2% of patients in the placebo group demonstrated a positive Outcome Measures in Rheumatology-OA Research Society International (OMERACT-OARSI) response at Week 4 [10], suggesting that placebo can have a significant therapeutic effect [11]. In one meta-analysis, the placebo response might account for about 75% of response to drugs commonly used in OA [12]. Boosting the intrinsic placebo response in OA treatment might improve clinical care. For this, it might be important to identify factors associated with a susceptibility to placebo effect [13]. However, it is unclear which patients with hand OA will benefit most from this placebo effect. In this post-hoc analysis of two RCTs, we aimed to identify factors associated with a clinically meaningful placebo response in patients with hand OA.

## Methods

### Study design

**Fig. 1:**
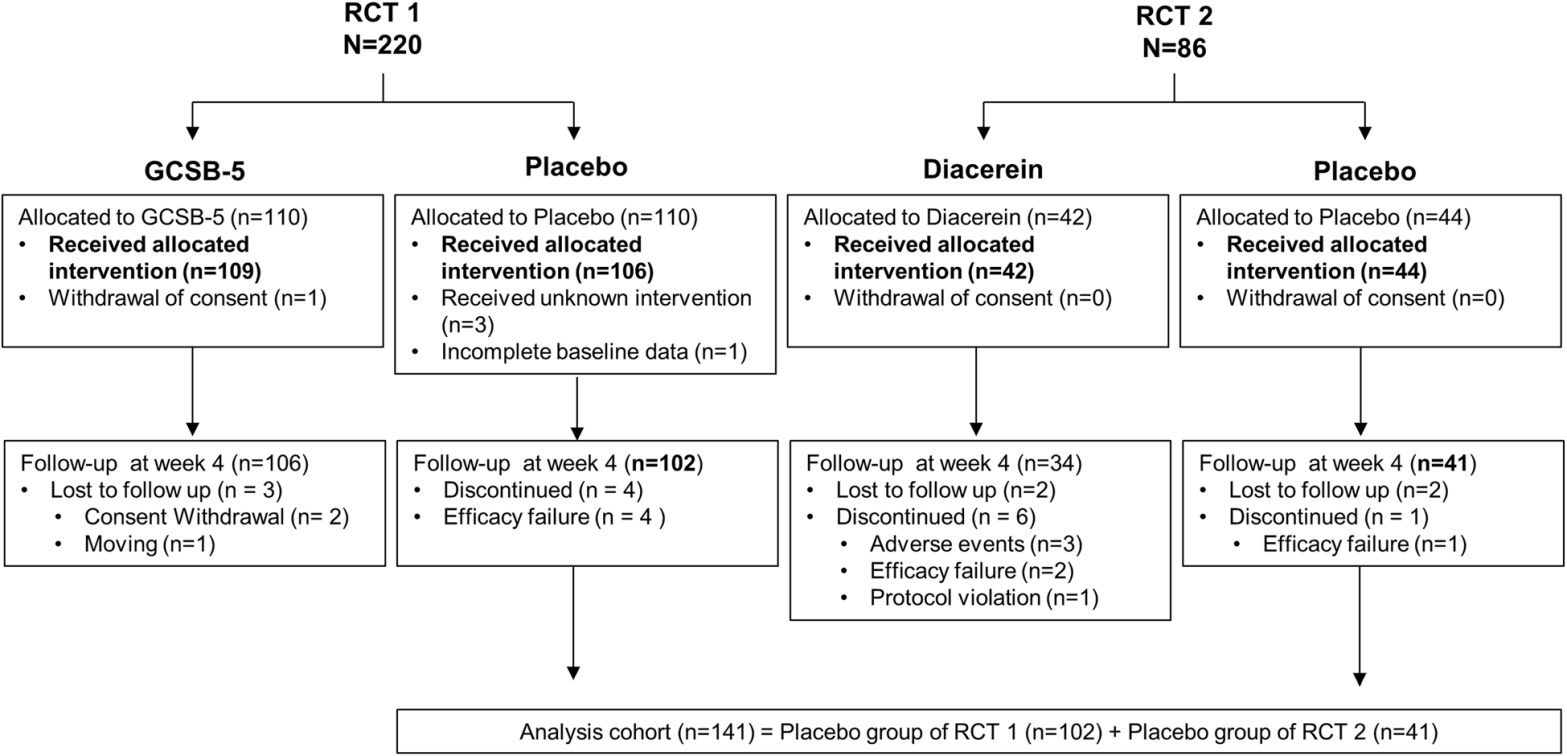
Flow diagram. RCT, randomized controlled trial

This post-hoc analysis was based on clinical and laboratory data from two prospective, double-blind, randomized, placebo-controlled trials designed to investigate the efficacy and safety of GCSB-5 or diacerein for treating hand OA; both studies were conducted in accordance with the Declaration of Helsinki [10, 14]. In the first RCT, 220 patients with hand OA according to the 1990 American College of Rheumatology (ACR) criteria for hand OA [15], all of whom were aged > 40 years and had pain exceeding 30/100 mm on a visual analog scale in the preceding 48 h, were randomly assigned to receive oral GCSB-5 (600 mg) or placebo twice a day for 12 weeks [10]. In the second RCT, 86 patients with hand OA according to the 1990 ACR for hand OA were randomized to receive diacerein (50 mg) or placebo twice a day [14]. All participating patients provided written informed consent. The study was approved by the institutional review boards of all participating centers and was registered at ClinicalTrials.gov (study no: NCT01910116 and NCT00685542). The post-hoc analysis included 102 patients with hand OA that were in the placebo group of the first RCT and 41 patients that were in the placebo group of the second RCT; patients with available clinical and laboratory parameters at baseline and at Week 4 were included in the analysis group (*n* = 143) (Fig. 1).

### Outcome

The efficacy endpoints included changes in the following variable from baseline: the AUSCAN pain score (0–100), the AUSCAN stiffness score (0–100), the AUSCAN function score (0–100), a patient global assessment (0–100), a physician global assessment (0–100), and the OMERACT-OARSI response criteria. A clinically significant improvement in pain was defined as an improvement in the AUCAN pain score of 10 (0–100) or more [16]. Patients deemed to be OMERACT-OARSI responders when they showed an improvement relative to baseline in pain or function domains of ≥50% with an absolute change of ≥20, or an improvement relative to baseline in at least two of three (pain, function, and patient global assessment) domains of ≥20% with an absolute change of ≥10 [17].

### Statistical analysis

An independent t-test and the Chi-squared test or Fisher’s exact test (as appropriate) were used to compare placebo responders and non-responders in terms of demographics and clinical variables. Normality of variables was examined using Kolmogorov-Smirnov test. Correlations between pain and clinical parameters were assessed using Pearson’s correlation. Multivariable logistic regression analysis was performed to identify factors associated with a clinically meaningful response. *P* < 0.05 was considered to indicate statistical significance. All analyses were performed by using IBM SPSS Statistics 22 software. All statistical analyses were performed by the authors.

## Results

### Patients’ characteristics

The mean age of the 102 patients with hand OA in RCT 1 and 41 patients in RCT 2 were 59.4 ± 8.0 years and 61.7 ± 18.9 years, respectively. Women were dominant in both RCTs. The mean disease duration of patients in RCT 1 and those in RCT 2 were 31.5 ± 47.7 months and 60.4 ± 61.1 months, respectively. Baseline characteristics of patients including AUSCAN pain, stiffness and function score were comparable between both groups (Table 1).

**Table 1:** Baseline characteristics of patients with hand OA who received placebo in the two randomized controlled trials

Baseline characteristics	RCT 1 (*n* = 102)	RCT 2 (*n* = 41)	*p*-value
Age, years	59.2 ± 8.0	61.7 ± 18.9	0.305
Female, n (%)	95 (93.1)	39 (95.1)	0.496
Weight, kg	59.1 ± 8.0	60.4 ± 8.9	0.398
Height, cm	157.0 ± 6.0	156.0 ± 6.3	0.348
Body mass index, kg/m^2^	23.9 ± 2.9	24.7 ± 2.8	0.129
Duration of hand OA, months	31.5 ± 47.7	60.4 ± 61.1	**0.009**
Baseline			
AUSCAN pain score (1–100)	47.8 ± 19.8	48.9 ± 25.9	0.788
AUSCAN stiffness score (1–100)	60.6 ± 21.7	63.9 ± 27.6	0.504
AUSCAN function score (1–100)	45.7 ± 23.7	41.7 ± 27.1	0.382
Patient global assessment (1–100)	49.6 ± 15.9	60.8 ± 19.4	0.002
Physician global assessment (1–100)	41.0 ± 13.0	42.6 ± 10.5	0.471
Tender joint count	6.3 ± 5.1	5.5 ± 5	0.400
Swollen joint count	0.9 ± 2.4	0.0 ± 0.3	0.000
Palpable node count	5.2 ± 2.5	N/A	
Deformed joint count	2.0 ± 1.7	N/A	
Erythrocyte sedimentation rate, mm/hr	12.9 ± 9.4	16.1 ± 11.6	0.086
hs-CRP, mg/dL (normal < 0.5 mg/dL)	0.12 ± 0.26	0.21 ± 0.76	0.317
Prior treatment, n (%)		N/A	
Acetaminophen/Tramadol	10 (9.8)		
Acetaminophen	1 (1.0)		
NSAIDs	36 (35.3)		
Glucosamine	13 (12.7)		
Diacerein	3 (2.9)		
Others	3 (2.9)		

Data are presented as the mean (SD) or n (%). *AUSCAN* Australian/Canadian Osteoarthritis Hand Index; *CRP* C-reactive protein, *ESR* Erythrocyte sedimentation rate, *N/A* Not available, *NSAID* Non-steroidal anti-inflammatory drug, *OA* Osteoarthritis, *RCT* Randomized controlled trial.

### AUSCAN pain

The mean AUSCAN pain score at baseline was 47.8 ± 19.8 in RCT 1 and 48.9 ± 25.9 in RCT 2 (Table 1). In RCT 1, the AUSCAN pain score was associated with the AUSCAN stiffness score (*r* = 0.312, *p* < 0.001), the AUSCAN function score (*r* = 0.743, *p* < 0.001), the patient global assessment (*r* = 0.393, *p* < 0.001), and the physician global assessment (*r* = 0.205, *p* = 0.039). However, the AUSCAN pain score was not associated with the tender joint count (*r* = 0.057, *p* = 0.567), the swollen joint count (*r* = 0.032, *p* = 0.749), the enlarged joint count (*r* = − 0.044, *p* = 0.659), or the deformed joint count (*r* = − 0.065, *p* = 0.515) at baseline. Similar correlations between baseline AUSCAN pain and other clinical characteristics were observed in RCT 2, except for TJC, which correlated with baseline AUSCAN-pain (*r* = 0.506, *p* = 0.001) (Supplementary Table S1).

### Factors associated with a significant placebo response

**Fig. 2:**
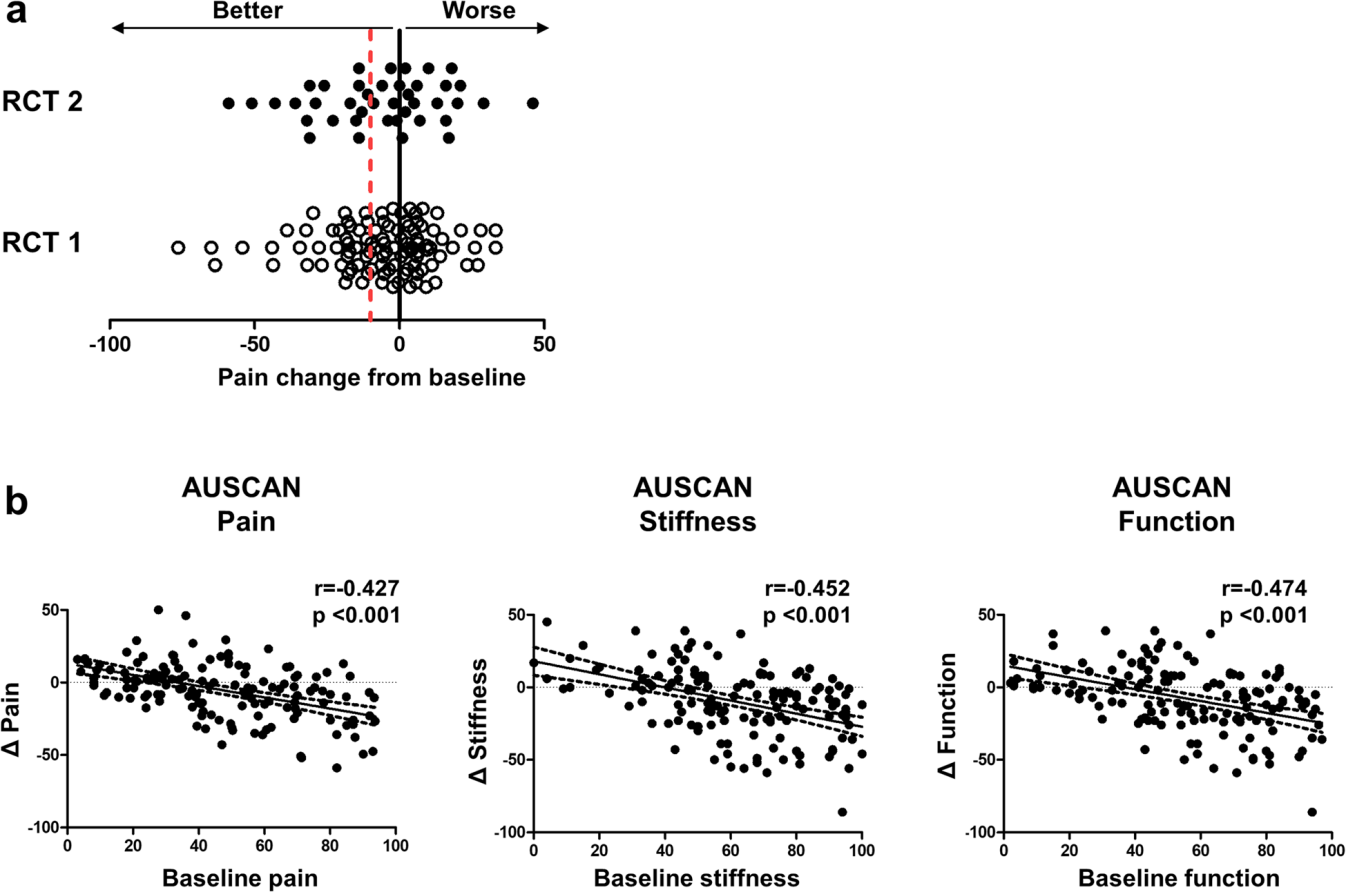
Placebo response. **a** Change in AUSCAN-pain at 4 weeks from the baseline in 102 patients in RCT 1 and 41 patients in RCT 2 were depicted. Red dotted line marks the clinically meaningful response. **b** Correlation between pain, stiffness, and function at baseline and placebo responses in 143 patients with hand osteoarthritis. Scatterplot represents the relation between the change in pain, stiffness and function and their respective baseline value. Correlations were examined by using Pearson’s correlation analysis

The overall improvement in the AUSCAN pain in 143 patients was − 6.0 ± 20.3. The mean improvement in the AUSCAN pain score did not differ between RCT 1 and RCT 2 (− 6.0 ± 19.7 vs. -6.1 ± 22.1, *p* = 0.944). The change in pain varied markedly between patients, ranging from − 76.4 to 33.2 in RCT 1 and − 59 to 46.0 in RCT 2 from baseline (Fig. 2a). Patients in RCT 1 and RCT 2 who received placebo showed a similar response with respect to improved pain, stiffness, and function scores. In addition, change in patient and physician global assessments at week 4 were similar between RCTs.

At 4 weeks, 54 (37.8%) of the 143 patients showed a clinically meaningful improvement (i.e., pain reduction > 10) (Table 2). These patients had a worse AUSCAN pain score at baseline (55.7 ± 19.7 vs. 43.6 ± 21.6, *p* = 0.001), a worse AUSCAN stiffness (68.2 ± 20.5 vs. 57.5 ± 24.5, *p* = 0.008) than patients without clinical improvement. The tender joint count (TJC), the swollen joint count (SJC), the enlarged joint count, and the deformed joint count did not differ between patients with or without clinically meaningful improvement. More patients showing clinically meaningful improvement used tramadol-AAP at baseline than those not showing clinical improvement (18.9% vs. 1.5%, respectively; *p* = 0.034). There was no difference between groups with respect to other medications, including NSAIDs and glucosamine (Table 2).

**Table 2:** Demographic and clinical characteristics of the 143 hand OA patients in randomized controlled trial 1 and 2 according to clinically significant improvement

	Response (−) (***n*** = 89)	Response (+) (***n*** = 54)	***P***-value
Age, years	58.1 ± 7.5	60 ± 8.2	0.164
Female	81 (91.0)	53 (98.1)	0.153
Weight, kg	59.1 ± 8.3	59.9 ± 8.3	0.606
Height, cm	157.3 ± 5.9	155.8 ± 6.2	0.150
BMI, kg/m^2^	23.9 ± 2.7	24.7 ± 3.1	0.105
OA duration, month	3.2 ± 4	3.5 ± 5.1	0.657
AUSCAN Pain	43.6 ± 21.6	55.7 ± 19.7	**0.001**
AUSCAN Stiffness	57.5 ± 24.5	68.2 ± 20.5	**0.008**
AUSCAN Function	41.7 ± 23.2	49.1 ± 26.6	0.083
Patient GA	51.1 ± 16	55.6 ± 19.9	0.158
Physician GA	40.1 ± 11.3	43.7 ± 13.6	0.084
Tender JC	5.8 ± 4.7	6.5 ± 5.7	0.426
Swollen JC	0.8 ± 2.4	0.5 ± 1.5	0.328
Enlarged JC ^a^	5.1 ± 2.7	5.4 ± 2.2	0.622
Deformity JC ^a^	2 ± 1.6	1.9 ± 1.9	0.658
ESR	0.17 ± 0.52	0.09 ± 0.13	0.291
CRP	13.4 ± 10.4	14.5 ± 9.6	0.533
Prior treatment			
Tramadol-AAP ^a^	3 (4.6)	7 (18.9)	**0.034**
Tramadol ^a^	1 (1.5)	0 (0)	1.000
NSAIDs ^a^	22 (33.8)	14 (37.8)	0.685
Diacerin ^a^	2 (3.1)	1 (2.7)	1.000
Glucosamine ^a^	9 (13.8)	4 (10.8)	0.765
Others ^a^	1 (1.5)	2 (5.4)	0.297

Data are presented as the mean (SD) or n (%). *P* values were generated by using an independent t-test (continuous variables) or the Chi-squared test (categorical variables). ^a^ Data were not available in the placebo group of RCT 2. *AUSCAN* Australian/Canadian Osteoarthritis Hand Index, *CRP* C-reactive protein, *ESR* Erythrocyte sedimentation rate, *NSAID* Non-steroidal anti-inflammatory drug, *OA* Osteoarthritis. Joints according to ACR OA classification criteria were evaluated.

Strikingly, there was a correlation between improvement in pain and level of pain at baseline (Pearson *r* = − 0.427, *p* < 0.001). In addition, change in stiffness and function from baseline correlated with baseline stiffness (Pearson *r* = − 0.425, *p* < 0.001) and baseline function (Pearson *r* = − 0.474, *p* < 0.001), respectively (Fig. 2b).

### Factors associated with a clinically meaningful placebo response

A logistic regression analysis was performed to identify factors associated with a clinically meaningful placebo response. In a univariable analysis, baseline AUSCAN pain (OR [95% CI] 1.028, [1.0105–1.0458], *p* = 0.002) and baseline AUSCAN function (1.021 [1.005–1.0371], *p* = 0.010) were associated with a better placebo response. In a multivariable analysis, only baseline AUSCAN pain was associated with clinically meaningful placebo response (1.054 [1.019–1.089], *p* = 0.002).

## Discussion

This post-hoc analysis of two prospective, double-blind, randomized, placebo-controlled studies shows that placebo yielded a clinically meaningful improvement in about one third of patients with hand OA. This placebo response was associated significantly with baseline pain, but not with structural changes such as joint swelling or osteophyte formation.

Hand OA is common, with a prevalence ranging from 29 to 76% [1, 18]. In half of patients, the disease will progress, leading to severe functional limitation and a serious disease burden [19]. In the absence of effective DMOADs, symptoms (i.e., pain, function and stiffness) are controlled by NSAIDs, tramadol, and opioid analgesics. However, the potential gastrointestinal and cardiovascular side effects of these drugs limit long-term use [20–23].

Although pain associated with OA is caused by structural changes due to accelerated degeneration of articular cartilage and secondary bone remodeling, pain signals are ultimately perceived by the brain after intensive central pain processing at multiple levels [24]. Consistent with this, we found that pain at baseline was not associated with structural changes such as swollen joints, nor was it associated with osteophyte formation and joint deformity (Supplementary Table S1). Pain correlated with the tender joint count only in RCT 2. Rather, pain was more closely associated with subjective parameters such as the AUCAN stiffness and function scores. Similarly, improvements in AUSCAN stiffness and function scores correlated with baseline stiffness and function, respectively. Taken together, not only pain generation in joints, but also central pain processing, might ultimately determine the level of pain and functional impairment experienced by patients with hand OA.

Although the mean improvement in AUSCAN pain was low, pain responses to placebo varied markedly among the OA patients, ranging from − 76.4 - 33.2 from baseline. Strikingly, high baseline pain, but not the severity of structural joint changes, was associated with a better placebo response (Table 1) [25]. This is consistent with a prior observation demonstrating that neither structural damage observed on ultrasound nor clinical severity of OA are predictive of treatment response [26]; this further supports the dissociation between treatment response and structural joint changes in those with hand OA. Rather, we found that improvements in pain, function, and stiffness correlated significantly with their respective baseline levels (Fig. 1).

While baseline AUSCAN pain and function were associated with a clinically meaningful placebo response, the multivariable analysis identified only the baseline AUSCAN pain as the factor for the placebo response. Interestingly, women with hand OA were 10 fold more likely to have a positive placebo response (Table 3), consistent with sex difference in the placebo response [27]. While OA affects both men and women, women were dominant in both RCTs, consistent with female dominance in the recent trials with hand OA [6, 9]. This suggests that women might suffer more from hand OA than men and they, therefore, are more likely to seek medical attention. Whether women with hand OA are more susceptible to pain, placebo response or both needs further investigation.

**Table 3:** Factors associated with a clinically meaningful placebo response

	Univariate			Multivariate		
Variables	OR	95% CI	***P*** value	OR	95% CI	***P*** value
Age	1.031	0.987–1.077	0.164	1.039	0.987–1.094	0.141
Gender (female)	5.235	0.636–43.068	0.124	10.552	0.931–119.633	0.057
Weight, kg	1.011	0.970–1.053	0.603			
Height, cm	0.958	0.902–1.016	0.152	1.010	0.934–1.091	0.811
BMI	1.104	0.979–1.245	0.108	1.104	0.969–1.258	0.136
Ds duration	1.017	0.944–1.097	0.655			
AUSCAN-Pain	1.028	1.010–1.046	**0.002**	1.054	1.019–1.089	**0.002**
AUSCAN-Function	1.021	1.005–1.037	**0.010**	0.974	0.947–1.001	0.058
AUSCAN-Stiffness	1.028	0.998–1.027	0.423			
Physician global assessment	1.025	0.996–1.054	0.088	1.018	0.986–1.052	0.270
Patient global assessment	1.015	0.995–1.035	0.137	0.998	0.972–1.024	0.872
Tender JC	1.028	0.961–1.098	0.423			
Swollen JC	0.914	0.762–1.097	0.334			
CRP	0.444	0.08–2.474	0.355			
ESR	1.011	0.978–1.045	0.531			

Multivariate logistic regression was performed. Variables that showed association (*p* < 0.2) in the univariable analysis were included in the multivariable analysis. *AUSCAN* Australian/Canadian Osteoarthritis Hand Index, *BMI* Body mass index, *CI* Confidence interval, *CRP* C-reactive protein, *Ds* Disease, *ESR* Erythrocyte sedimentation rate, *JC* Joint count, *NSAID* Non-steroidal anti-inflammatory drug, *OR* Odds ratio, *OA* Osteoarthritis.

Placebo effect is not limited to hand OA and it depends on the mode of delivery. In knee OA, intra-articular and topical placebo elicited a greater placebo response than oral placebo [28]. The placebo effect can vary among OA sites since it was greater in knee OA than in hip OA [29]. Taken together, all placebo are not equal. However, it is still important to identify additional factors associated with a treatment response to optimize clinical care of OA patients. As example, early radiographic features such as congruent articular reduction and tiabial plateau alignment were associated with a better pain improvement after surgical treatment of displaced tibial plateau fractures [30]. It is interesting that use of tramadol/AAP was also associated with a better placebo response, whereas NSAIDs and other medications were not. Tramadol acts on central pain processing; it is a weak agonist of the mu opiate receptor and inhibits both serotonin and norepinephrine reuptake, thereby exerting anti-nociceptive effects [31].

A previous study shows that in patients with chronic pain and associated pain sensitization (such as those with fibromyalgia), the retention rate for tramadol/AAP is higher than that for placebo [32, 33]. Therefore, OA patients with severe pain might have developed aberrant central pain processing over time, resulting in increased central sensitization [34]. OA patients who were taking tramadol/AAP at baseline might benefit from anti-nociceptive effects on central pain processing, making them more susceptible to placebo effects. Indeed, duloxetine, which modifies central pain sensitization, is an effective treatment for knee OA [35]. The mechanism underlying central pain processing in OA requires further investigation.

It might be unethical to prescribe a placebo in routine clinical practice. However, the inherent placebo effect of any pharmacological and non-pharmacological treatment could be optimized in routine clinical practice. This is of particular importance since the placebo response might account for about 75% of response to drugs that are commonly used in OA treatment [12]. To optimize this placebo effect, it might be crucial to identify patients who are more susceptible to a placebo response. In this study, female gender and high baseline pain were associated with a clinically significant placebo response (Table 3). The question of whether a warm and reassuring consultation, optimistic attitudes of healthcare providers, and positive relationships between patient and physician improve OA outcomes should be investigated in future.

This study has several limitations. First, the lack of a control group that did not receive any treatment (even placebo) makes estimating the placebo effect difficult. Second, we did not consider depressive mood disorders and/or emotional or physical stress, which might influence pain processing and so placebo responses. Third, RCT 2 (41 patients in the placebo arm) is too small to enable comparison of clinical parameters between clinical responders and non-responders. However, both RCT 1 and RCT 2 showed remarkably similar placebo responses (Supplementary Table S2). Further studies are needed to identify therapeutic and situational factors that improve placebo responses.

## Conclusions

The placebo effect can be significant in patients with hand OA who have high pain levels at baseline. Further studies are needed to understand the pathophysiology and underlying mechanisms, and to optimize the placebo effect as an OA treatment in clinical practice.

## Supplementary Information

**Additional file 1: Table S1.** Correlation between AUSCAN pain with baseline characteristics. **Table S2.** Placebo response at 4 weeks in RCT 1 and RCT 2.

## Data Availability

The data that support the results of this study are available from the corresponding author upon reasonable request.

## Abbreviations

ACR: American College of Rheumatology
AUSCAN: Australian/Canadian Osteoarthritis Hand Index
DMOADs: Disease-modifying osteoarthritis drug
NSAID: Non-steroidal anti-inflammatory drug
OA: Osteoarthritis
OMERACT-OARSI: Outcome Measures in Rheumatology-OA Research Society International
RCT: Randomized trial
RA: Rheumatoid arthritis
SJC: Swollen joint count
TJC: Tender joint count
