# Supplementary material for: Predictors of a placebo response in patients with hand osteoarthritis: post-hoc analysis of two randomized controlled trials
Source: BMC Musculoskelet Disord. 2021 Mar 4;22:244. doi: 10.1186/s12891-021-04089-9 (PMC7934539; doi:10.1186/s12891-021-04089-9)
Supplement: Supplementary file 1 — Additional file 1: Table S1. Correlation between AUSCAN pain with baseline characteristics. Table S2. Placebo response at 4 weeks in RCT 1 and RCT 2. [file 12891_2021_4089_MOESM1_ESM.pdf]

SUPPLEMENTARY MATERIAL

Supplementary table S1. Correlation between AUSCAN pain with baseline characteristics.

**AUSCAN-pain**		**Age**	**OA duration**	**BMI**	**Weight**	**Height**	**CRP**	**ESR**	**TJC**	**SJC**	**EJC**	**DJC**	**AUSCAN-Stiff**	**AUSCAN-Fct**
**RCT 1**	**r**	-0.023	-0.107	0.071	0.069	-0.010	-0.139	-0.022	0.057	0.032	-0.044	-0.065	0.312	0.743
	**p**	0.815	0.284	0.477	0.489	0.918	0.165	0.822	0.567	0.749	0.659	0.515	**0.001**	**<0.001**
**RCT 2**	**r**	0.192	0.197	0.074	-0.007	-0.114	0.184	-0.143	0.506	-0.129	N/A	N/A	0.658	0.835
	**p**	0.229	0.217	0.644	0.963	0.478	0.339	0.377	**0.001**	0.421	N/A	N/A	**<0.001**	**<0.001**

Correlation between pain and other characteristics was calculated using Pearson's correlation.

AUSCAN, Australian/Canadian Osteoarthritis Hand Index; CRP, C-reactive protein; DJC, deformed joint count; Fct, function; EJC, enlarged joint count; ESR, erythrocyte sedimentation rate; NSAID, non-steroidal anti-inflammatory drug; OA, osteoarthritis. SJC, swollen joint count; TJC, tender joint count; Stiff, stiffness. N/A, not available.

Supplementary table S2. Placebo response (change from baseline) at 4 weeks in RCT 1 and RCT 2.

	RCT 1			RCT 2			
	**Baseline**	**Week 4**	**Difference**	**Baseline**	**Week 4**	**Difference**	**P-value***
AUSCAN pain	47.8 ± 19.8	41.8 ± 21.8	-6.0 ± 19.7	48.9 ± 25.9	42.8 ± 24.5	-6.1 ± 22.1	0.788
AUSCAN stiffness	60.6 ± 21.7	51 ± 24.5	-9.6 ± 23.9	63.9 ± 27.6	53.8 ± 25.2	-10.0 ± 23.2	0.504
AUSCAN function	45.7 ± 23.7	41.1 ± 21.9	-4.5 ± 18.4	41.7 ± 27.1	39.8 ± 25.1	-1.8 ± 18.8	0.382
Patient global assessment	49.6 ± 15.9	44.6 ± 21.6	-5.0 ± 19.6	60.8 ± 19.4	51 ± 26.4	-9.7 ± 20.7	**0.002**
Physician global assessment	41.0 ± 13.0	30.9 ± 14.1	-10.1 ± 15.6	42.6 ± 10.5	33.6 ± 15	-8.8 ± 11.9	0.471
Tender joint count	6.3 ± 5.1	5.3 ± 5.4	-1.0 ± 4.4	5.5 ± 5	5.3 ± 5.6	-0.3 ± 4.5	0.400
Swollen joint count	0.9 ± 2.4	0.5 ± 1.8	-0.4 ± 2.2	0.0 ± 0.3	0.1 ± 0.5	0.0 ± 0.6	**<0.001**

AUSCAN, Australian/Canadian Osteoarthritis Hand Index; RCT, randomized controlled trial.
***Comparison of difference (change from baseline) between RCT 1 and RCT 2.**
